# Is it feasible to learn research skills in addition to audit skills through clinical audit? A mixed methods study in general practice

**DOI:** 10.1007/s11845-021-02802-0

**Published:** 2021-10-19

**Authors:** Crea Carberry, Ian Callanan, Geoff McCombe, Helen Tobin, Gerard Bury, Jason Last, Walter Cullen

**Affiliations:** 1 School of Medicine, University College Dublin, Dublin, Ireland; 2 Clinical Audit Facilitator, St. Vincent’s Healthcare Group, Dublin, Ireland

**Keywords:** Clinical audit, Curriculum design, General practice, Medical education, Undergraduate research skills

## Abstract

**Background:**

Involving medical students in research in their undergraduate careers may increase the likelihood that they will be research active after graduation. To date, there has been a paucity of published research of students doing research in general practice.

**Aim:**

The study aims to evaluate the impact of general practice clinical audits on early-stage graduate entry students’ audit and research self-efficacy and explore feasibility issues from the student and GP perspective.

**Methods:**

Two student questionnaires (pre- and post-intervention), a qualitative GP survey of the 25 participating GPs and semi-structured interviews of a purposeful sample of GPs were conducted.

**Results:**

Participating students who completed the follow-up survey found that it had a positive educational impact (55%), increased their understanding of the audit cycle (72%) and real-world prescribing (77%). Research confidence wise, there was a statistically significant difference in the student group who completed the audit project compared to those students who did not in knowledge of the audit cycle and the difference between research and audit (*p* = 0.001) but not in other research skills. Ninety-six percent of responding GPs would be happy for students to do future audits in their practice but some feasibility issues similar to other research initiatives in general practice were identified.

**Conclusion:**

We found this audit initiative feasible and useful in helping students learn about audit skills, patient safety and real-world prescribing. GPs and students would benefit more if it were linked to a substantial clinical placement, focussed on a topic of interest and given protected time. Separate research projects may be needed to develop research skills confidence.

## Introduction

There is a decreasing number of physician scientists at a time when there is an increased demand for evidence-based medicine and research [1–3]. Medical schools have a key role to play in this regard, as studies have shown that involving medical students in active research in their undergraduate careers may increase the likelihood that they will be research active after graduation [4–6].

In 2012, The Association for Medical Education in Europe (AMEE) produced a guide (*’Developing research skills in medical students’*) which recommended that every medical student must understand research methods and the benefits that research brings to their profession [7]. This guide concluded by stating that understanding of research can be greatly enhanced by encouraging the active participation by students in research activities.

The Medical Education in Europe (MEDINE) Thematic Network had previously undertaken a pan-European consensus survey to generate a set of research competencies and core learning outcomes for medical curricula in Europe across all three Bologna cycles (Bachelor, Master and Doctor). These research-specific learning competencies can be broadly considered in three groups: ‘generic’ competences, those related to ‘using research’ and those related to ‘doing research’. The MEDINE network conducted a Delphi exercise, attempting to identify key research skills to include in curricula, known as the MEDINE2 survey [8]. For this study, we looked at ‘doing research’ options as outlined by MEDINE which would correlate with the World Federation for Medical Education (WFME)’s 2015 quality improvement standard in relation to research.

Quality development standard: The medical school should in the curriculum include elements of original or advanced research. Elements of original or advanced research would include obligatory or elective analytic and experimental studies, thereby fostering the ability to participate in the scientific development of medicine as professionals and colleagues [9].

In the 2016 Medical Schools Council/Health Education England publication in relation to promoting general practice careers amongst medical students ‘*Not by chance, by choice*’, recommendation 13 states that ‘All institutions influencing students should collaborate to raise the profile of academic general practice by ensuring all students have access to, and are overtly valued and rewarded, for scholarly activity and visibly supervised by primary care leads’ [10]. This study looks to specifically examine one option to engage students in elements of original research in general practice, getting first-year graduate entry medical students to do a clinical audit as part of their general practice placements. It was hoped that through audit, students would learn in a systematic way about key research areas such as sampling, data collection/analysis (simple descriptive statistics), dissemination of knowledge and reflective practice in addition to audit skills.

To date, there has been a paucity of published research of students doing research in general practice. There have been some mainly descriptive papers describing initiatives to promote medical student research in primary care/general practice/family medicine [11–14].

In 2014, Mullan et al. looked at medical student self-perceived research experiences pre- and post-community research projects in an integrated research curriculum and reported a statistically significant improvement in nine out of the ten research areas [15].

There have been several papers which have discussed the possibility of using intercalated degrees to potentially increase interest in research in Primary Care by medical students. Creavin et al. described their experience of running a pilot intercalated Masters in Primary Care, an intercalated MPhil [16], and Jones et al. discussed an intercalated BSc in Primary Care from the student and feasibility perspective [16–18].

The audit option has been previously reported in general practice [19–24] but not in the context of measuring medical student pre- and post-audit and research skill self-efficacy and looking at student and GP feasibility issues as objectives. Chapman et al. in 2015 looked at research self confidence in students pre and post a clinical audit (these audits were done in a surgical setting, not general practice). The authors reported increased confidence in data collection in a clinical setting (*p* < 0.001) and presentation of scientific results (*p* < 0.013). Collaborators also reported an increased appreciation of research, audit and study design (*p* < 0.001) [25].

Feasibility and acceptance issues of medical students performing a ‘research task’ (documentation of potential drug interactions with statins) on general practice placements were discussed by Moßhammer et al. in 2016 [26]. They found that the overall assessment of the project by the students was on average ‘satisfactory’ and differed from the assessment by the teaching physicians which was rated as ‘good’. This study aimed to look at student research self-efficacy/self-perceived research and audit experience pre and post the audit initiative as well as student/GP feasibility issues and to compare findings to those of Mullan, Chapman and Moßhammer.

## Aims

The study aims to evaluate the impact of general practice clinical audits on students’ audit and research self-efficacy and explore feasibility issues from the student and GP perspective.

## Methods

**Fig. 1:**
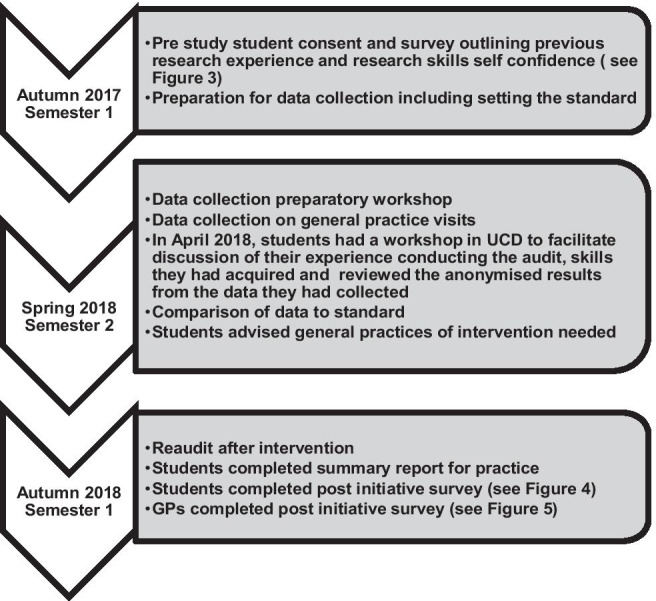
Overview of audit initiative

This mixed methods study involved two student questionnaires (pre- and post-audit initiative), a GP survey after the initiative and semi-structured interviews of a purposeful sample of participating GPs. When qualitative and quantitative methods are used in combination, the strengths of each lead to a better understanding of the research questions [27]. See Fig. 1 for overview of the audit initiative.

### Description of audit initiative

#### Semester 1 autumn 2017

The audit process ran over a 15-month period from September 2017 to December 2018. At the outset, a series of lectures was given to first-year graduate entry medical students outlining the purpose of the activity, the theory of clinical audit, data protection, aspects of professionalism (especially confidentiality) and practical requirements and considerations. Students/GPs then decided whether to opt into this extracurricular initiative (see “Recruitment” section). All students were assigned to one of four audit topics aligned to the cardiorespiratory module they would be doing in spring 2018. As they were first-year students, it was decided to give students topics aligned to their curriculum rather than give the students/GPs choice in their audit topic.

In small groups, students had to review the evidence behind the standard which was at the core of the clinical audit and did group presentations about their findings in autumn 2017.

#### Semester 2 spring 2018

**Fig. 2:**
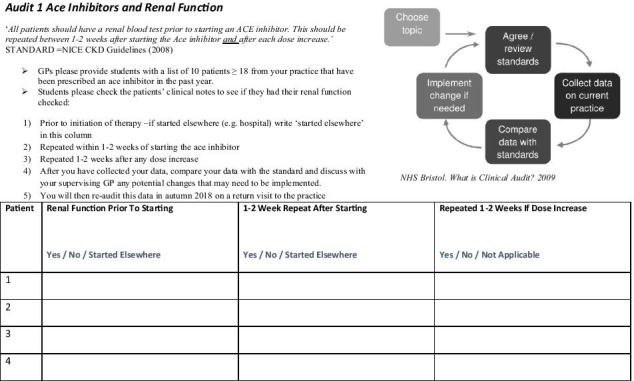
Sample data collection sheet

In January 2018, students had a preparatory workshop in a Computer-Assisted Lab in which they practised reviewing dummy patient notes and extracting data using one of the GP record keeping software programmes (Socrates®). A handbook was provided for the GPs and students in preparation (available on request). The students then undertook data collection for the first round of the audit cycle in spring 2018 (see Fig. 2 for a sample data collection sheet).

Students then had a review session with the academic team in which they discussed their experience conducting the audit, skills they had acquired and reviewed anonymised results from the data they had collected. This was done in a large group setting. After the review session with UCD academic staff, they contacted their assigned general practices to discuss their findings and any suggested interventions.

#### Semester 1 autumn 2018

Students did their re-audit in autumn 2018 in their assigned practices and were advised to produce a confidential, anonymised report for the GP practice. Academic staff at UCD did not have access to these reports. A review session was held with the academic staff and students completed the follow-up student survey.

### Recruitment

As this was a feasibility study, students and GPs were invited to take part in the study. Participation in the study was on a voluntary basis with no extra academic credit for students or extra financial compensation for GPs. It was effectively ‘extra homework’ for both GPs and students.

#### Students

The student cohort selected for this study were the 2017 intake of graduate entry medical students (104 students). An information session outlining the programme and inviting them to participate was held in September 2017.

#### General practitioners

General practitioner tutors on the UCD GP Network were introduced to the initiative at a GP study day and were then invited via letter to participate.

### Type of surveys

Paper surveys were distributed to both the GPs and students using ‘Sphinx Survey’ ® compatible hard copy surveys and then converted to Excel files using Sphinx software. It was decided to use hard copy surveys rather than electronic surveys because previous experience had found in general an increased response rates for surveys with paper-based surveys [28, 29]. The surveys were converted from Excel ® files to SPSS Version 24.0 ®.

### Student surveys

#### Instrument

A pre- and post-study questionnaire was adapted with permission from the STARSurg 2015 initiative/MEDINE 2 consensus survey of research competencies [25, 30]. The Medical Education in Europe (MEDINE) Thematic Network had previously undertaken a pan-European consensus survey to generate a set of research competencies and core learning outcomes for medical curricula in Europe across all three Bologna cycles (Bachelor, Master and Doctor). These research-specific learning competencies can be broadly considered in three groups: ‘generic’ competences, those related to ‘using research’ and those related to ‘doing research’. The MEDINE network conducted a Delphi exercise, attempting to identify key research skills to include in curricula, known as the MEDINE2 survey [8]. For this survey, the ‘doing research’ options as outlined by MEDINE were looked at which would correlate with the World Federation for Medical Education (WFME)’s 2015 quality improvement standard in relation to research. Bee and Murdoch Eaton’s previously published guidelines on questionnaire development was also consulted [31].

The surveys contained questions pertaining to the students’ prior research experience and paired research confidence pre- and post-initiative (whether they had done an audit or not). Ethical approval was granted to include the last four digits of students’ numbers on the pre- and post-student surveys so that students could be matched to check for change in their research confidence over the year. These four numbers were then changed to a different number — ‘student 1’, ‘student 2’, etc.

The post-survey also contained questions on their experience of the initiative if they had taken part and feasibility questions for those who had not taken part/withdrew from the initiative. All students were also asked to document any other research initiatives they had participated in over the year.

**Fig. 3:**
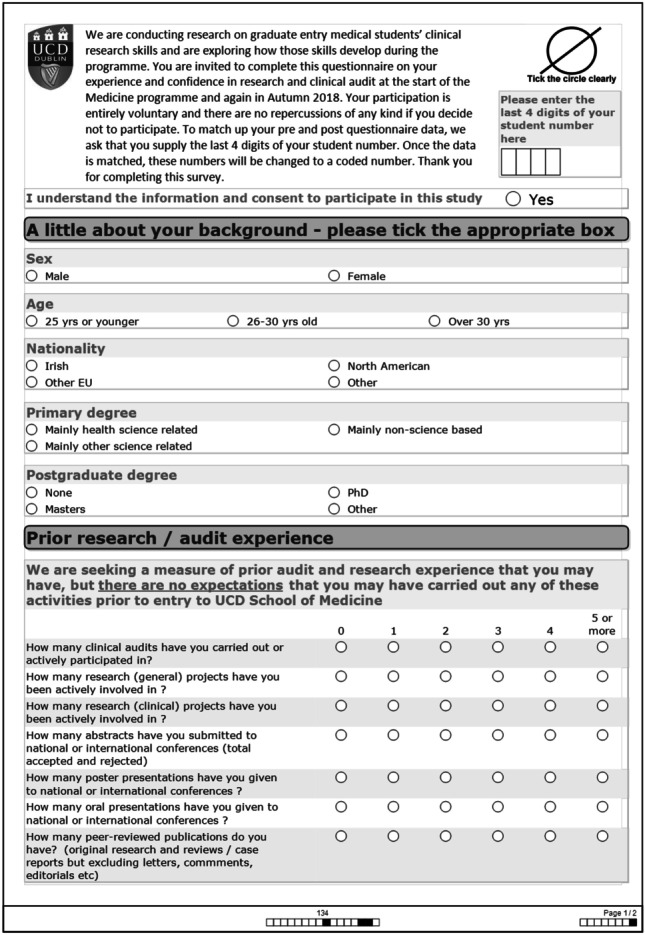

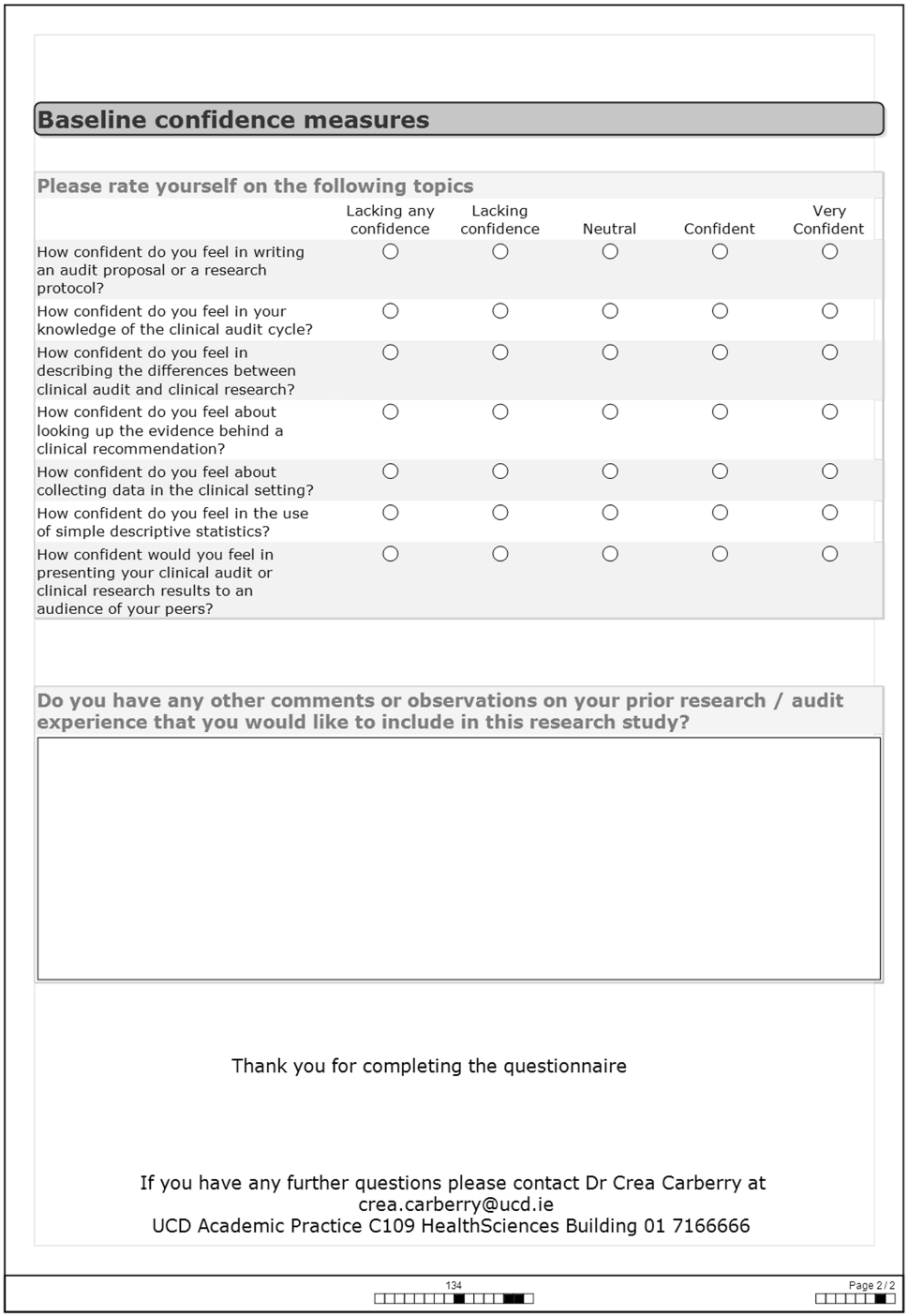
Student pre-study survey

**Fig. 4:**
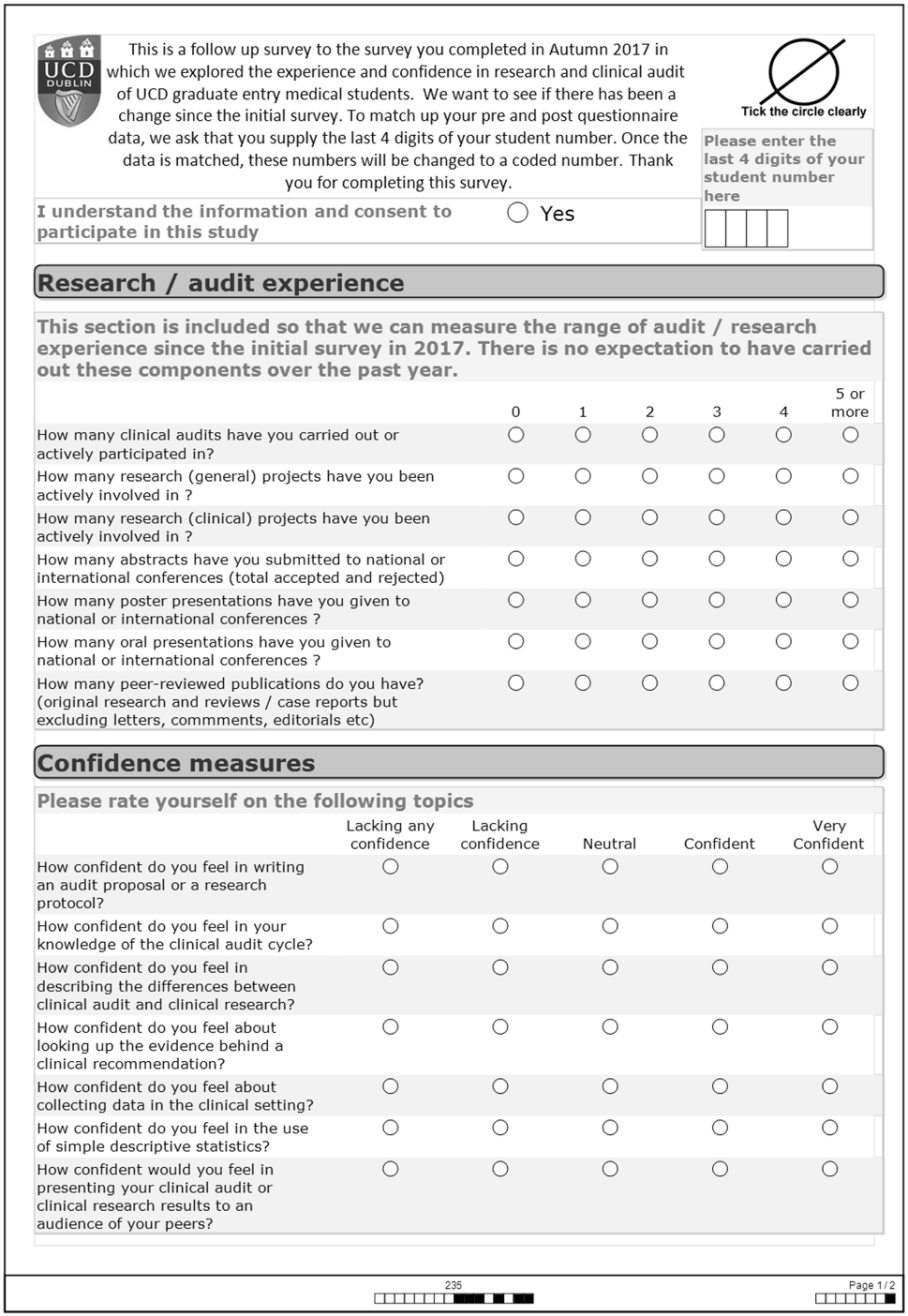

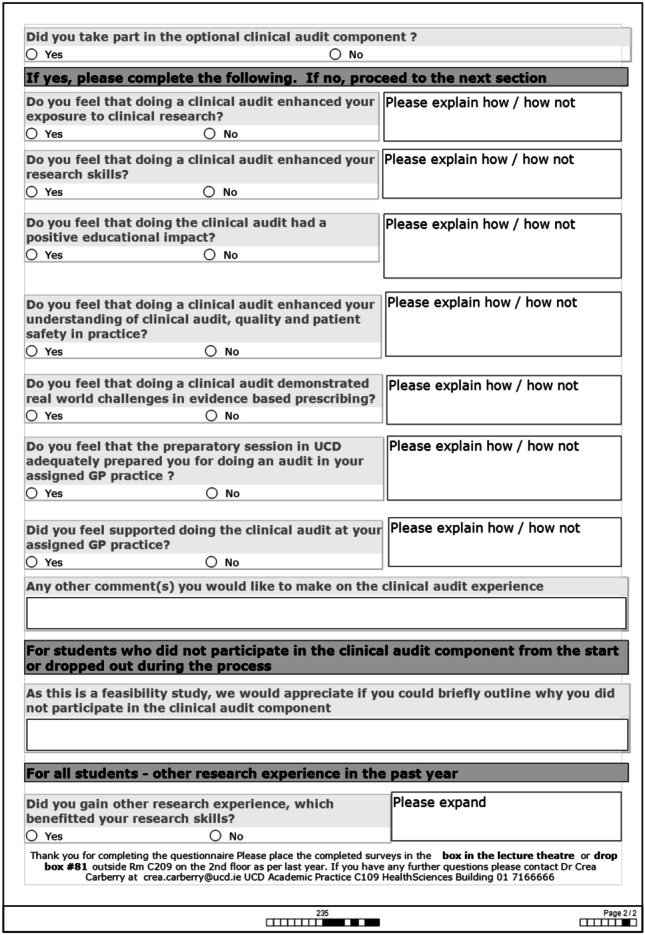
Student post-study survey

The surveys were converted from Excel ® files to SPSS Version 24.0 ® and variables coded. To find out whether there was a significant difference in the responses, Wilcoxon signed ranks test was used when the same groups were compared before and after and for groups that were not related to each other but were independent, the non-parametric test for independent groups, Mann–Whitney *U*, was used when there were two categories and Kruskal–Wallis for more than two categories (see Figs. 3 and 4 for student surveys).

### GP survey

**Fig. 5:**
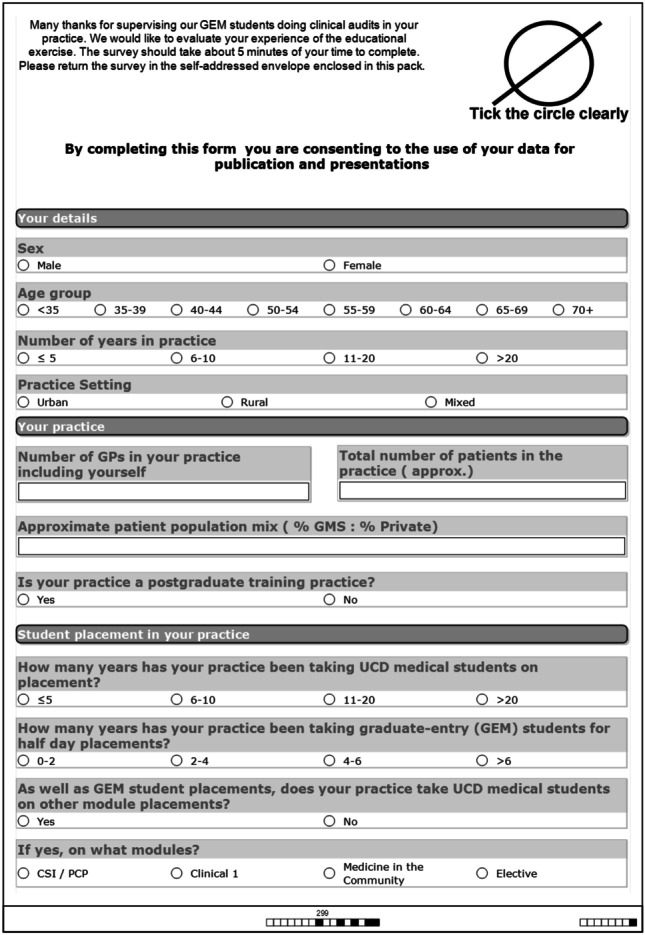

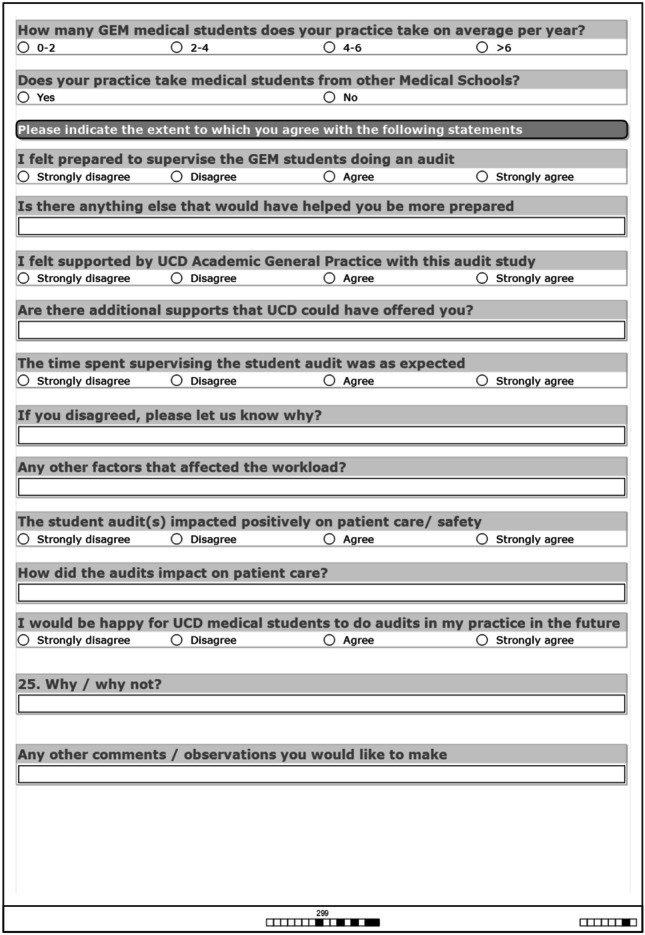
GP survey

Paper surveys were distributed to the 25 participating GPs. The demographics of questions were based on categories used in a national survey of general practice in Ireland *conducted periodically from 1982 to 2015* [32] (see Fig. 5).

### GP semi-structured interviews

We conducted telephone semi-structured interviews.

**Fig. 6:**
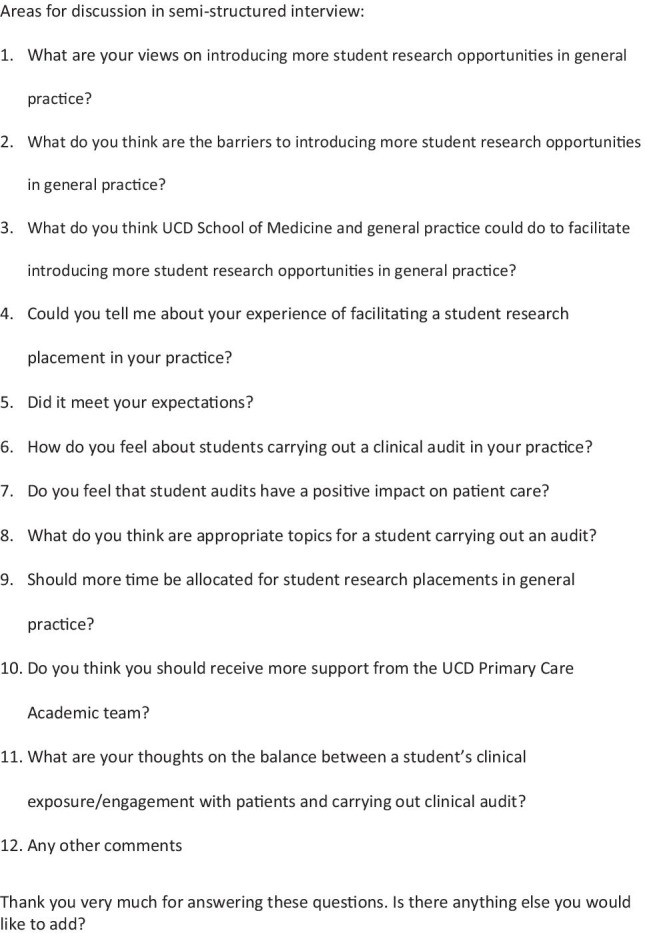
GP semi-structured interview content

We had planned to conduct a focus group for the qualitative work with GPs but due to restrictions as a result of the COVID-19 pandemic, this was cancelled. In notifying GPs of this, we asked if they would be willing to participate in a telephone interview instead. Those who indicated they would, were sent the written information in advance, signed the consent form and the telephone interview was conducted at a time that was convenient for them. Ethical approval was granted for this change. The interviews were conducted by a member of the research team who is an experienced qualitative researcher and had not been involved in programme delivery. Content for the GP interviews was developed from findings of the GP survey and literature review (see Fig. 6 for GP interview content).

The GPs were sent a summary of the findings of the qualitative survey prior to the interviews. The telephone interviews were audio recorded, transcribed and thematic analysis using Braun and Clarke’s framework was used to analyse the transcripts [33] (see Table 5).

## Results

### Student component

#### Participation-student numbers opting in to do audit

1. As it was a feasibility study, students were given the choice of opting in to participate in the audit component on their GP placements. In September 2017, 92/104 students opted in for this component.
2. In January 2018, prior to training workshops being delivered and data collected, 14 students withdrew from the initiative with heavy workload being the most commonly cited reason for withdrawal. One student had withdrawn from the Degree Programme and two had been unwell and so had not participated. In May 2018, another student withdrew stating that partner had withdrawn from the programme and cited difficulty contacting the GP in order to complete the placement. Therefore, 74 students started doing audits in the general practices.

#### Research experience and confidence questionnaire

1. Pre-study questionnaire: 96% response rate, 100/104 students completed the survey outlining their previous research/audit experience and research skill confidence (see Tables 1, 2, and 3).
2. Post-study questionnaire: 59% response rate, 58/99 students completed the post-study questionnaire (five students from the original 104 student cohort did not progress to the next year for various reasons).

**Table 1:** Student demographics pre-study

**Sex**	58% were female, 42% male
**Age**	82% percent of students were < 25 years old (82%), 15% were 26–30 years and 3% > 30 years
**Nationality**	60% of the student cohort were Irish, 24% North American, 12% other EU (including UK) and 4% other nationalities
**Primary degree**	56% of students had a primary degree in health science or other science (29%), 15% had a non-science related primary degree
**Postgraduate degree**	Most students had no postgraduate degree (79%) with 19% having a master’s degree, 1% a PhD and 1% classified as ‘other

**Table 2:** Student research experience pre-study

**Prior audit**	97% had not done a prior clinical audit
**Prior general project**	19% had not done any prior general projects with the majority having done one project (40%). 4% of students had completed five or more general research projects
**Prior clinical projects**	74% had not completed any clinical projects with 3% having completed three or more
**Prior abstract submission**	78% had not submitted an abstract to a national or international conference. 1% had submitted to five or more conferences
**Prior poster presentation**	80% had not had a poster presentation at a national or international conference. 2% had five or more poster presentations at national or international conferences
**Prior oral presentation**	90% had not done an oral presentation at a national or international conference. 2% had done two or more oral presentations
**Prior peer review publication**	79% had no peer reviewed publications with 1% having three or more peer reviewed publications

**Table 3:** Median research confidence score pre-study

**Confidence**	**Before**
How confident do you feel in your knowledge of the clinical audit cycle?	**3**
How confident do you feel describing the differences between clinical audit and research?	**2.5**
How confident do you feel in looking up the evidence behind a clinical recommendation?	**3**
How confident do you feel in writing an audit proposal or research protocol?	**4**
How confident do you feel collecting data in a clinical setting?	**3**
How confident do you feel in the use of simple descriptive statistics?	**4**
How confident do you feel presenting results to peers?	**4**

#### Prior research experience

Most students did not have much research experience at the start of the graduate entry programme (see Table 2).

#### Research self-confidence/efficacy at the start of medical school

Students self-rated their research confidence in various research tasks using a Likert scale from 1 to 5 (where 1 = lacking any confidence and 5 = very confident). It can be seen that the lowest median score for any task was 2.5 (this was for describing the difference between research and clinical audit). The median score for each task is listed in Table 3 below.

#### Post-study questionnaire results of 58/99 students (this survey was offered to all students whether they took part in the audit initiative or not)

Seventy-two percent of these respondents had taken part in the audit initiative and 28% had not. Forty-five percent of the students had done other research over the year (for example summer student research projects).

Overall, although some increases, especially in those who had now done a clinical audit, there was not much change in student research output.

#### Research experience after 1 year

1. Seventy-four percent of respondents had now completed a clinical audit (compared to 97% who had not 1 year previously).
2. Fourteen percent had still not done any general projects with the majority having done one project (compared to 19% who had not 1 year prior).
3. Fifty-two percent had not completed any clinical projects (compared to 74% who had not 1 year prior).
4. Seventy-four percent had not submitted an abstract to a national or international conference (compared to 78% who had not 1 year prior).
5. Sixty-seven percent had not had a poster presentation at a national or international conference (compared to 80% who had not 1 year prior).
6. Eighty-one percent had not done an oral presentation at a national or international conference (compared to 90% who had not 1 year prior).
7. Seventy-nine percent had no peer-reviewed publications (no change to the previous year).

#### Change in research self-confidence after 1 year

On the follow-up survey, we were able to match up 51 students to see if their research/audit self-confidence had increased since their entry into UCD Medicine.

Text box 1. Changes in research confidence after 1 year in matched students 1. There was a statistically significant difference in students’ self confidence in writing a research proposal in the 51 matched students (whether they had taken part in the audit initiative or not) (*p* = 0.048 using Wilcoxon test).

2. For the group who completed the audit, there was increased student confidence in the knowledge of the audit cycle and the difference between research and audit (*p* = 0.001 for both using Wilcoxon test).

3. There was a statistically significant difference in confidence in writing a research proposal in those who had a health science degree compared to those who had a different science degree/non-science degree (*p* = 0.047 using Kruskal–Wallis) and students who had a non-science degree appeared to gain confidence in simple descriptive statistics, but otherwise, there was no difference between change on research confidence based on primary degree type.

4. There was no statistically significant difference in research confidence after 1 year in those with post-grad degrees (masters/PhD) versus those that had primary degree only for any of the research skills (Mann–Whitney test used).

5. There was not any statistically significant difference in research confidence after 1 year based on age/sex/nationality.

#### Student satisfaction/feedback on initiative

For those who took part in the audit initiative and responded to the post survey, there was mixed feedback on the initiative. Overall, they did find it had a positive educational impact (55% answered yes to this), increased their understanding of the audit cycle (72.4%) and real-world prescribing (77%) but they did not find it increased their research exposure or research skills (only 29% felt it enhanced their research skills). Overall, they felt they were adequately prepared to do an audit by the academic team in UCD (71%) and they felt supported by the GPs in the general practices (80%). There were several feasibility issues mentioned; lack of time/workload, no academic credit, issues to do with GP/role modelling, issues with assigned student partner and issues with assigned topic (see Table 4).

**Table 4:** Feasibility issues for students—themes from survey open questions

**Lack of time/workload**	‘Limited time during the school year.’ ‘Busy lifestyle. Hard to find time.’ ‘Was concerned it would take up too much time.’
No academic credit	‘Prioritisation of aspects of the course carrying credits.’ ‘There was no grade for the audit and so I did not want to do extra work.’
Issues to do with GP/role modelling	‘The GP felt that the audit was a waste of time. They said that they were aware of guidelines but did not have time to implement them. They were not very receptive to making changes and as a result we did not re-audit.’ **‘**Our GP was not too enthusiastic in the audit first round, like did not continue due to our own time constraints.’
Issues with assigned student partner	‘My partner in the audit didn’t return after the summer so the initial data collection was not sufficient (I didn’t realise he wasn’t coming back)’. ‘Partner dropped out first.’
Issues with assigned topic	‘I also thought the topic of my audit (LFTS with statins) had been demonstrated in the literature to be evidence based. I would have liked to have had a choice of topic before having to do the intervention.’ ‘My audit topic was checking inhaler technique, however the GP informed us that they never record this.’
Previous research experience	‘Had previous research experience, didn’t feel necessary.’
*Regrets about not opting in*	‘Did not realise importance of audit in continuing development and CV building. Would have done it with understanding I have now.’

### GP survey

Twenty-five GPs in the Dublin area agreed to have the students undertake audits in their practices. There was a 96% response rate from participating GPs (24/25).

#### Demographics of participating GPs

Fifty-four percent were male and 48% female. Ten (42%) of GPs were aged 50–64 years old with only three GPs aged less than 39 and two GPs aged over 65. Ninety-two percent of practices were group practices. Sixty-seven percent of practices had between 2000 and 6000 patients. Twenty-nine percent of participating practices were postgraduate training practices. Seventy-five percent of practices had been taking UCD medical students for > 5 years with four practices (17%) having UCD medical students for > 20 years. The median number of GEM students per practice was 34.

#### Statistically significant results from GP survey

1. Sex: male GPs were more likely to have GEM students do audits in their practice in the future than female GPs (*p* = 0.044 using Mann–Whitney test).
2. Years in practice: those in practice 10–20 years disagreed that the workload was as expected (*p* = 0.041 using Kruskal–Wallis).
3. Post-graduate training practice versus non post-graduate training practice: those from non-training practices did not feel as supported by UCD (*p* = 0.009 using Mann–Whitney test).
4. Single-handed versus group practice: those from single-handed practices felt more prepared to supervise student audits’ (*p* = 0.026 using Mann–Whitney) and that the initiative had a positive impact on patient care (*p* = 0.026 using Mann–Whitney). However, there were only two GP practices in the single-handed category.

*No statistically significant difference (using Kruskal–Wallis) in GP age/number of patients in practice/years taking UCD medical students/number of GEM students doing placements.

#### GP satisfaction with initiative

1. Seventy-one percent of the GPs felt that the student audits had a positive impact on patient care.
2. Eighty-seven percent of the responding GPs felt they were supported by the academic team with the audits.
3. Ninety-six percent of responding GPs (23/24) said they would be happy for UCD students to do future audits in their practice but one GP reported that they would not be happy to support future students doing audits in their practice. This GP felt that student clinical exposure and interaction with patients should be prioritised over audit participation.

#### GP feasibility issues identified in survey

1. The main area that the GPs felt needed working on with the initiative was the time allocated — GPs felt more time needed to be allocated.
2. At times there was a discrepancy in the topics the GPs wanted audited and the topics that had been assigned.

### GP semi-structured interviews

The three themes that featured most commonly were the following:

1. GP feasibility issues
2. Student interest in doing research
3. The balance between time spent on research and time spent on clinical learning during general practice placements (see Table 5)

**Table 5:** Themes from GP semi-structured interviews

*Theme 1: GP feasibility issues*
**(a) Time**
‘The students then themselves once they have time set aside, because they can obviously set aside a number of days or half days when they are not involved clinically in the practice and they have the time set aside when they can actually go ahead and do the audit of course. Whereas we would struggle because of clinical commitments.’ (GP 5) ‘I suppose like anything, it is time constraints both on the students and the GPs behalf. (GP 1)
**(b) Space/computer access/software**
‘…we actually physically we don’t have the space. We have just got a registrar so we are bit more hemmed in…. The problem is that being students, we could not have them working remotely with the patient data. I want everything to stay on site and they walk home with nothing.’ (GP 4) ‘I think probably rooms and computers, so from a resource perspective that you would have a spare room for a student to be able to sit at the computer and carry out some research.’ (GP 1)
**(c) GDPR issues/data protection concerns**
One GP had concerns with this while the others did not as they had policies in place ‘I suppose finding the barrier of GDPR. I am not fully sure that I would understand the extent of GDPR and students and audit, and how the three interact.’ (GP 1) ‘Well, we do have a sample GDPR patient confidentiality form that we do get the medical students to all sign and it is stored in our GDPR folder. To be honest, we do have a chat with them before they start about the confidentiality issue. But to be honest, they have all done, that has all been explained to them already in the university. I know that they do modules on that before they go out to the surgeries, isn’t that correct?’ (GP 4)
**(d) GP audit/research/audit knowledge**
‘Perhaps the GPs might need some tuition on the audit cycle as well as the students. Perhaps it is assumed that GPs understand audit but that might not be the case.’ (GP1) ‘I think they have somewhat more experience than me in doing audits per se. It is only something that we have been doing in latter years since the Irish Medical Council would have mandated it in the college. I think one of the traits of GPs is that they maybe steer clear of research and audit because it wasn’t part of our post graduate training. Certainly, when I was qualifying in year XXXX, we went the hospital route and a requirement to do audits and some research where it wasn’t a requirement at our time.’ (GP 5) GP IT skills/IT issues I think how IT savvy and how research savvy, all those things within the tutor is probably a predictive factor for how good, how well the audit might go for the student.’ (GP 1)
**(e) Trusting/delegation**
‘Yeah, it would be a lovely idea to think that they could do the audit or be involved in it. But I know, personally, for me, it is me who has to do everything because I am the boss. So, with Covid-19 it is me who does absolutely every single thing for all my staff, all of the time. Nobody else does it. If I had students come in and I would have to talk to them all about audits, I have to talk to every other member, every other doctor here my audit as well. I am saying, “Oh, my God, another thing that I have to do”.' (GP 2)
**(f) Choice of topic**
Two GPs felt that the students/GPs should be given a choice in the topic while the other three GPs did not have a preference ‘Because I think, as I said, I think that might just generate a topic that might be of more interest to both parties and therefore might make it more doable in the long run. So, I think a bit more autonomy in the project’s outline would be my first suggestion.’ (GP 1) ‘…and then perhaps maybe outline the projects that they would see as being achievable — more maybe to create their own ideas from.’ (GP 1) ‘I suppose the first consideration is whether or not it is mandatory for students to perform research and if so, does it have to be in general practice. But I suppose I am wondering would there be a richer output of research if the students had a choice or who they wanted to pair up with to do their research. I think a certain portion of them would choose general practice naturally.' (GP 1)
*Theme 2: student engagement/ability to do audit*
There were mixed responses to how well the GPs felt the students engaged with the initiative and the students’ ability to do the audit. One GP felt that the three student pairs who were assigned to their practice lost interest while another thought that all students were very interested and able ‘I suppose the interest levels were highest at the start and waned. Which I think would be typical of most research projects, but it seemed to happen quite quickly with the students. I suppose the two things that struck me was number one, was that it was optional, so I think they were very interested at the start. I think when they thought it might boost their CV but when they realised that they didn’t need it for their core curriculum, they were inclined to put that to the bottom of the pile and lost interest.’(GP 1) ‘And I mean, they educate us as well as us trying to help them, but they definitely try to help us. So, it is a two-way thing. They are brilliant.’ (GP 3) ‘They are quite good at actually going through the charts and applying the tests that they are meant to apply just to see. Also, I mean, the older students definitely, the mature ones, they understand context.’ (GP 4)
***Theme 3: the balance between time spent on research and time spent on clinical learning during general practice placements***
Four out of the five GPs felt that clinical interactions/clinical learning should be prioritised over time spent on research ‘So, I think overall it would be that students could perform research in general practice but for the specific students that I met on that particular placement it seemed to be that it was probably too heavy on research, if that makes sense.’ (GP 1) ‘But personally, I think they need more clinical time as opposed to research time. I think research time is really valuable down the line but now in GP, we do our own audits every year, so we are much more used to them. It is a great learning but they have to have the clinical skills. It is so much more important as a base.’ (GP 2)

## Discussion

### Summary

The main findings of this initiative from the medical student perspective were that they found it had a positive educational impact, increased their understanding of the audit cycle and real-world prescribing but did not find doing an audit in general practice increased their research exposure or research skills/confidence. Overall, they felt they were adequately prepared to do an audit by the academic team in UCD and they felt supported by the GPs in the general practices. Parallel to the audit initiative, 45% of the respondents had engaged in other research activities; over the year however there was not much of an increase in research output. Research confidence wise, there was a statistically significant difference in the group who completed the audit in their confidence in the knowledge of the audit cycle and the difference between research and audit (*p* = 0.001) but no statistically significant difference in other research skills or difference based on demographics/whether they had a postgraduate degree. There were several feasibility issues mentioned: lack of time/workload, no academic credit, issues to do with GP/role modelling, issues with assigned student partner and issues with assigned topic.

### Comparison with existing literature

The increased awareness of the difference between research and audit by medical students was also found by Chapman et al. in their 2015 study looking at research confidence pre- and post-surgical audits. Chapman et al. also found an increased confidence in data collection in a clinical setting (*p* < 0.001) and presentation of scientific results (*p* < 0.013) which we did not find [25]. In 2014, Mullan et al. reported an improvement on medical student self-perceived research experiences pre- and post-community research projects in an integrated research curriculum. In their study, they had a higher response rate in the follow-up test, and although measuring some different research skills, they reported statistically significant improvements in nine (out of the ten) areas. In their study, students did a research project as part of a much longer community placement (12 months) [15].

#### Student feasibility issues

Student feasibility issues encountered included time constraints in a busy curriculum, issues with the assigned topic and GP supervisor issues. Issues with the assigned topic and supervisor were also reported by students in Moßhammer’s 2016 paper looking at feasibility and acceptability of a short research task on 2-week general practice placements [26]. Time constraints and supervisor issues were also reported by medical students in Nottingham in Nikkar-Esfahani’s 2012 paper on medical student audit and research projects [34].

#### GP perspective

The key findings in relation to the participating GPs were that although 96% of the responding GPs (23/24) said they would be happy for students to do future audits in their practice and 71% of the GPs felt that the student audits had a positive impact on patient care, some feasibility issues were brought up — time constraints, space, getting the balance between time spent on clinical placement, audit/data protection training and choice of topic. These feasibility issues raised by the GPs were similar to findings of other research initiatives in general practice [35–39].

### Methodological considerations

Validated questionnaires were not found for the surveys; however, modified versions of previous questionnaires were found which enabled a comparison to be made to previous studies [25, 30].

We had planned to run a focus group for the qualitative work with GPs, but due to restrictions as a result of the COVID-19 pandemic, the focus group was cancelled and semi-structured interviews by telephone were carried out instead. The interviews were conducted by a member of the research team who is an experienced qualitative researcher and had not been involved in programme delivery. The advantages of this approach were health and safety, convenience, allowed GPs to participate and involved an interviewer who had not been involved in the programme delivery. The disadvantages were that interaction with the researcher/other GPs was not possible.

A possible confounding factor in relation to the change in students’ research self-efficacy over the year was that students could have acquired research skills doing other research projects over the year or had additional research teaching.

A limitation of the study was the dropout rate of students: 99/104 did the initial survey and 92/104 originally opted to do an audit. We documented why 18/92 did not start the audit. 58 out of 99 did the follow-up survey (five students left the programme).

### Implications for research, education, and practice ﻿policy

We found this audit initiative feasible and useful in helping students learn about audit skills, patient safety and real-world prescribing. Retention was a challenge. GPs and students would benefit more if it were linked to a substantial clinical placement; focussed on a topic that were of interest; linked to formal instruction in research skills, quality and safety; and given protected time.

Separate research projects may be needed to develop research skill confidence.

Pairing the opportunity to do research whilst on general practice placements may increase both interest in general practice and in research but more work needs to be done on this. Longitudinal primary care experiences are probably needed to achieve this [40].

## Data Availability

The datasets used and/or analysed during the current study are available from the corresponding author on reasonable request.
